# Free energy landscape of siRNA-polycation complexation: Elucidating the effect of molecular geometry, polymer flexibility, and charge neutralization

**DOI:** 10.1371/journal.pone.0186816

**Published:** 2017-10-31

**Authors:** Gianvito Grasso, Marco Agostino Deriu, Viorica Patrulea, Gerrit Borchard, Michael Möller, Andrea Danani

**Affiliations:** 1 Istituto Dalle Molle di Studi Sull'Intelligenza Artificiale (IDSIA), Scuola Universitaria Professionale della Svizzera Italiana (SUPSI), Università della Svizzera Italiana (USI), Centro Galleria 2, Manno, Switzerland; 2 School of Pharmaceutical Sciences, University of Geneva, Rue Michel-Servet 1, Geneva, Switzerland; Universidade Nova de Lisboa Instituto de Tecnologia Quimica e Biologica, PORTUGAL

## Abstract

The success of medical threatments with DNA and silencing interference RNA is strongly related to the design of efficient delivery technologies. Cationic polymers represent an attractive strategy to serve as nucleic-acid carriers with the envisioned advantages of efficient complexation, low cost, ease of production, well-defined size, and low polydispersity index. However, the balance between efficacy and toxicity (safety) of these polymers is a challenge and in need of improvement. With the aim of designing more effective polycationic-based gene carriers, many parameters such as carrier morphology, size, molecular weight, surface chemistry, and flexibility/rigidity ratio need to be taken into consideration. In the present work, the binding mechanism of three cationic polymers (polyarginine, polylysine and polyethyleneimine) to a model siRNA target is computationally investigated at the atomistic level. In order to better understand the polycationic carrier-siRNA interactions, replica exchange molecular dynamic simulations were carried out to provide an exhaustive exploration of all the possible binding sites, taking fully into account the siRNA flexibility together with the presence of explicit solvent and ions. Moreover, well-tempered metadynamics simulations were employed to elucidate how molecular geometry, polycation flexibility, and charge neutralization affect the siRNA-polycations free energy landscape in term of low-energy binding modes and unbinding free energy barriers. Significant differences among polymer binding modes have been detected, revealing the advantageous binding properties of polyarginine and polylysine compared to polyethyleneimine.

## Introduction

The development of molecular systems composed of nucleic acids and synthetic polymers is of great interest because of their application in gene therapy, which includes delivering genetic materials into cells for therapeutic purposes [1]. The negatively charged nucleic acids are not able to spontaneously cross the likewise negatively charged cell membrane, however, they may be ingested by the endo-/lysosomal pathway [2]. Therefore, the future success of medical treatments with DNA and silencing interference RNA (siRNA) in the gene therapy research field is strongly related to the design of efficient delivery technologies [3,4] that ensure the release from the endosomal pathway (endosomal escape). Several strategies have been developed in the past to achieve this goal. Despite viral vector-based strategies are widely used, resulting in efficient delivery and high transfection efficacy [5], their immunogenicity and potential severe side effects greatly limit their general use. Synthetic cationic polymers represent an attractive strategy to serve as carriers with the envisioned advantages of high stability, low cost, ease of production, well-defined size, and versatility for different applications [6]. At present, polyethylenemine (PEI) [7,8], polylysine (polyLYS) [9] and polyarginine (polyARG) [10,11], among others, have been investigated for gene delivery. The efficacy of PEI as gene delivery vector has been demonstrated to strongly depend on its structure, molecular mass [12] and charge density; however, significant cytotoxicity strongly limits its application [13]. Therefore, alternative cationic polymers such as polyARG or polyLYS have been widely considered and described in the literature. On one hand, polyLYS is characterized by good biodegradability and biocompatibility; on the other hand, its lack of enabling endosomal escape strongly impairs its transfection efficiency. Therefore the balance between the efficacy and toxicity of polycationic systems used for the safe and efficient delivery of DNA and RNA therapeutics still requires optimization [14]. With the aim of designing more effective polycationic-based gene carriers, many parameters need to be taken into consideration. These include carrier morphology, size, molecular weight, negative to positive (N/P) charge ratios between genetic material and carrier, surface chemistry, electrostatic potential, and flexibility/rigidity ratio. In this connection, computer simulations and in particular enhanced sampling techniques may be able to support the design of potent and selective nucleic acids carriers characterized by the best compromise between drug complexation stability and release ability of the delivery system [15–18].

A number of computational studies have demonstrated how the PEI architecture influences the interaction with DNA [19] together with the importance of degree of branching [20,21] and electrostatic attraction [22,23] in affecting the DNA/PEI binding. The importance of the free energy estimation in providing a molecular level understanding of the complexation between positively charged polymers and the negatively charged nucleic acids has been demonstrated [24]. The free energy landscape represents an effective index of the ability of the polycationic carrier to bind to the nucleic acid and also carries implications for the process of nucleic acid release within the cytosol. Unfortunately, the polycation unbinding process is typically a long time-scale event, in the order of microseconds to milliseconds, and therefore difficult to sample with standard techniques, such as classical molecular dynamics (MD). Thus, the use of the emerging enhanced sampling techniques such as metadynamics [25,26] or Replica Exchange Molecular Dynamics (REMD) [27–29] are of advantage. In the nucleic acids research field, metadynamics has been successfully used to study the transition from a biomolecule-DNA bound state, where the protein forms specific interactions with the base edges of the DNA molecule, to an unbound state where these specific interactions have been disrupted [30–33]. However, so far this technique has never been applied to investigate the binding of cationic polymers to nucleic acids as done in the present work.

Here the binding mechanism of three cationic polymers (polyARG, polyLYS and PEI) to an siRNA target was investigated at the atomistic level. REMD was carried out to provide an exhaustive exploration of all the possible binding sites, taking fully into account the siRNA flexibility together with the presence of explicit solvent and ions. Moreover, well-tempered metadynamics simulations were employed to elucidate how polymer flexibility, charge density, protonation ratio and molecular geometry affect the siRNA-polycations free energy landscape in term of lowest energy binding modes and unbinding free energy barriers. The results presented here have significant implications for the future design of potent and selective nucleic acids drug carriers to achieve the best compromise between complexation stability and release ability.

## Materials and methods

### System coordinates and topologies

**Fig 1:**
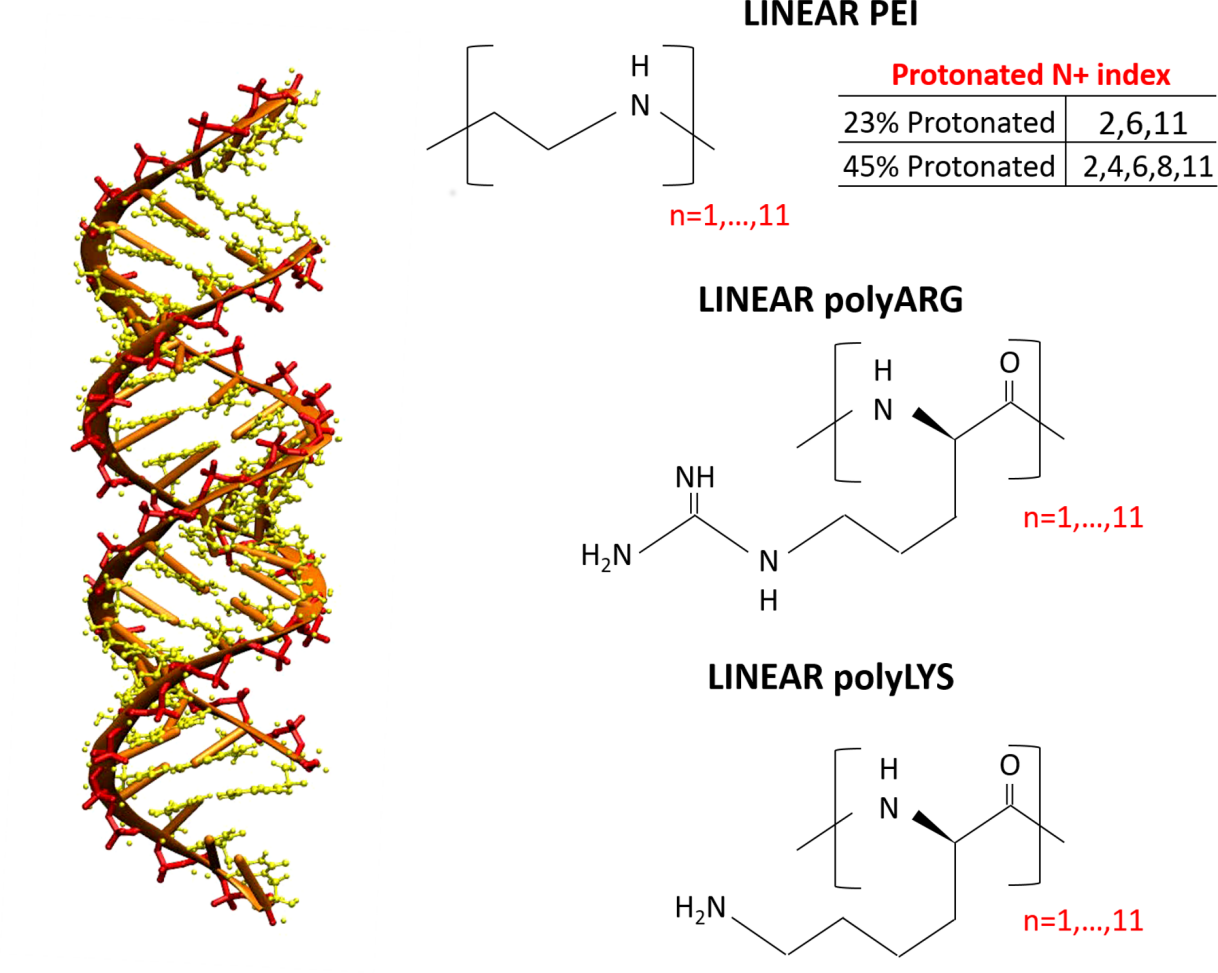
Structure of the siRNA sequence dGdG(AGCAGCACCUUCAGGAU)dUdU and the polycationic polymers investigated in the present work.

The siRNA sequence dGdG(AGCAGCACCUUCAGGAU)dUdU composed of 42 nucleotides, known for its activity in prostate cancer [34] was selected as a siRNA model for the present work. The starting coordinates were built to be a canonical B-form using the AMBER NAB tool. Four different polycationic polymers were chosen (Fig 1):

1. polyarginine with 10 repeating units (polyARG)
2. polylysine with 10 repeating units (polyLYS)
3. linear polyethylenimine with 10 repeating units and a protonation ratio of 27% (PEI27)
4. linear polyethylenimine with 10 repeating units and a protonation ratio of 45% (PEI45)

CHIMERA software [35] was used to build the PEI initial atomic structure. PEPFOLD-3 [36] was applied to predict the polyARG and polyLYS starting conformation. In the present work, we set up an initial protonation state of the system corresponding to a physiological pH 7.4. Under these conditions, each repeating unit of polyLYS and polyARG is protonated, whereas the protonation ratio of PEI amine groups has been a highly debated topic in literature [37–39]. However, most experimental and computational works reported a PEI protonation ratio in the range of 10% to 50% under physiological conditions [37–41]. For this reason, two different PEI protonation ratios were considered in the present work (27% and 45%). We assigned the protonation state of each amine group as uniformly as possible, considering that the uniform distribution of the protonation sites was theoretically confirmed [41].

The protonation state was set up at the beginning of the simulation. In this connection, it is worth mentioning that the treatment of pH effect is a complex issue in MD simulations. While simulating at a fixed pH, the dynamic change of the system protonation state should be allowed during the simulation. Several approaches have been applied with success to address this issue, such as Metropolis Monte Carlo [42–44], enveloping distribution sampling [45], or λ-dynamics [46–48]. However, this work follows the common practice in molecular dynamics [19–21,49,50] by defining the protonation state at the beginning of the simulation. Further developments of this work may consider to explicitly focus on the dynamic protonation/deprotonation at constant pH in polymer/siRNA complexes.

Considered polycations differ in both polymer geometry and protonation level (Fig 1). As all of the polymers consist of 10 repeating units, differences in their protonation state are related to the net charge of the polymer chain. In detail, the total charge of PEI27 and PEI45 amount to +3e and +5e, respectively. In turn, polyARG and polyLYS, completely protonated under physiological conditions, are characterized by a total charge of +10e. Moreover, the polyARG molecular geometry is characterized by the guanidinium group with three amine groups (two -NH_2_ and one–NH-) to attract and form H-bonds with oxygen atoms, while polyLYS has only has one such group (-NH_3_).

AMBER99-ILDN force-field [51–53] were chosen to describe the siRNA, polyLYS and polyARG topologies. The General Amber Force Field (GAFF) [54,55] and TIP3P model [56] have been employed for PEI and water molecules, as already done in previous work in literature [57]. Partial charges of PEI were obtained using the AM1-BCC method [58], widely used in the field of polymer partial charge calculation [59].

### Replica Exchange Molecular Dynamics

Each polycation chain was placed at 30 Å from the siRNA Center of Mass (COM) at the beginning of the simulations. The molecular systems (siRNA-PEI27, siRNA-PEI45, siRNA-polyLYS, siRNA-polyARG) were solvated in a cubic box in which the minimum distance between the protein and the edge of the box was 1.2 nm, resulting in a molecular system of about 40000 interacting particles. Each system was first neutralized by adding Cl− and Na+ ions (salt concentration of 0.15 M) and then minimized by 1000 steps of steepest descent energy minimization algorithm. Finally the system was equilibrated at 300 K [60] and 1 atm [61]. Replica Exchange Molecular Dynamics (REMD) [27] was carried out to efficiently sample the polycation-siRNA complex formation, following a computational procedure successfully applied in literature [28,62–65]. In detail, 128 replicas from 300 K to 530 K in NVT ensemble, were simulated for 50 ns, obtaining a cumulative simulation time of 6.5 μs for each system. Shorter simulation time has demonstrated to be effective in correctly describing the interaction between the polycationic chains and nucleic acids [19,49]. Simulated temperatures were distributed following the exponential spacing law as suggested in the literature [66,67], in order to obtain a constant overlap of the potential energy distributions among temperatures (S1.1 File). The exchange attempt time interval was set to 2 ps. At the end of the simulations, the resulting average exchange probability was 0.4. GROMACS 5.1.2 package was used for all MD simulations and data analysis [68]. Long-ranged electrostatic interactions were calculated at every step with the Particle-Mesh Ewald method with a cut-off of 1.2 nm. A cut-off of 1.2 nm was also applied to Lennard-Jones interactions. The LINCS algorithm [69] approach allowed an integration time step of 2 fs. The Visual Molecular Dynamics (VMD) [70] package was used for the visual inspection of the simulated systems. The nucleotides mainly responsible for siRNA-polycation interaction were identified by contact probability plots. Contact probability for each residue was calculated, as already described in literature [65,71], using the following procedure: for each snapshot of the MD trajectory at 300K, the distance between a nucleotide and the polycationic chain was calculated. If the distance value was equal or less than a chosen threshold (0.28 nm), the nucleotide was considered in contact with the interfacing monomer in that trajectory snapshot. The number of ‘‘contact snapshots” divided by the number of total snapshots taken out from the MD trajectories was the contact probability associated with the nucleotide.

### Metadynamics simulations

The free energy landscape representing the siRNA-polycationic chain interaction was investigated by means of Metadynamics [25,26], a powerful technique able to enhance sampling in MD simulations by adding to the total energy of the system a biasing history dependent term, obtained by the summation of Gaussian hills laying on the subspace identified by a set of user-defined Collective Variables (CVs) (22). The Gaussian hills are added at a constant time interval in the position explored by the system in the space of CVs. Starting from the most sampled region of the REMD trajectory at 300 K, the unbinding of each polycation from the siRNA system was simulated using Gromacs-5.1.2 [72] with the PLUMEDv2.3 patch [73]. Two CVs were considered for the calculation: the distance between the COM of the polycation from the major inertia axis of the target siRNA (distance from axis-DFA); the projection of the COM of the polycation on the major inertia axis of the target siRNA (projection on axis-POA). Along the simulation, a Gaussian deposition rate of 2 kJ/mol·ps was initially applied and gradually decreased on the basis of an adaptive scheme. The setting of Gaussian width and deposition rate was done on the basis of a well-established scheme [74,75]. In particular, the Gaussian width value was of the same order of magnitude as the standard deviation of the collective variable, calculated during unbiased simulations. Gaussian widths of 0.5 Å and 1 Å were used, for DFA and POA reaction coordinates, respectively. The reconstruction of the free-energy surface was performed by the reweighting algorithm procedure [76], allowing the identification of the main polycation binding sites together with the lowest energy conformations as function of four CVs:

- The distance between the COM of the polycation from the major inertia axis of the target siRNA (distance from axis-DFA)
- The projection of the COM of the polycation on the major inertia axis of the target siRNA (projection on axis-POA)

Each CV has demonstrated to be helpful to describe the binding events in the context of protein-nucleic acids complexes by metadynamics [30,31,33,77]. More specific information about the definition of the CVsS2), the reweighting algorithm procedure (S1.3 File) and the convergence of the Metadynamics simulations (S1.4 File) are reported in the Supporting Information text.

Each molecular conformation corresponding to the free energy well was considered as input for the electrostatic calculations. The electrostatic potentials were computed by the APBS package [78]. In detail, the non linear Poisson-Boltzmann equation was applied using single Debye-Huckel sphere boundary conditions on a 65x65x65 grid with a spacing of 1Å centered at the COM of the molecular system. The relative dielectric constants of the solute and the solvent were set to 4 and 78.4 [79,80], respectively. The ionic strength was set to 150 mM and the temperature was fixed at 300K [79,80].

## Results

We present our simulation results in three parts. First, REMD simulations were analyzed paying particular attention to the siRNA-polycation complex formation at atomistic level. Secondly, metadynamics simulations were employed to estimate the siRNA-polycation free energy landscape, elucidating the effect of molecular geometry, polycation flexibility and charge density on the polycation binding modes. Finally, the most representative polycation binding conformations were further investigated to describe the electrostatic potential and the charge neutralization mechanism of the resulting complex.

### The siRNA-polycation complexes at atomistic levels

**Fig 2:**
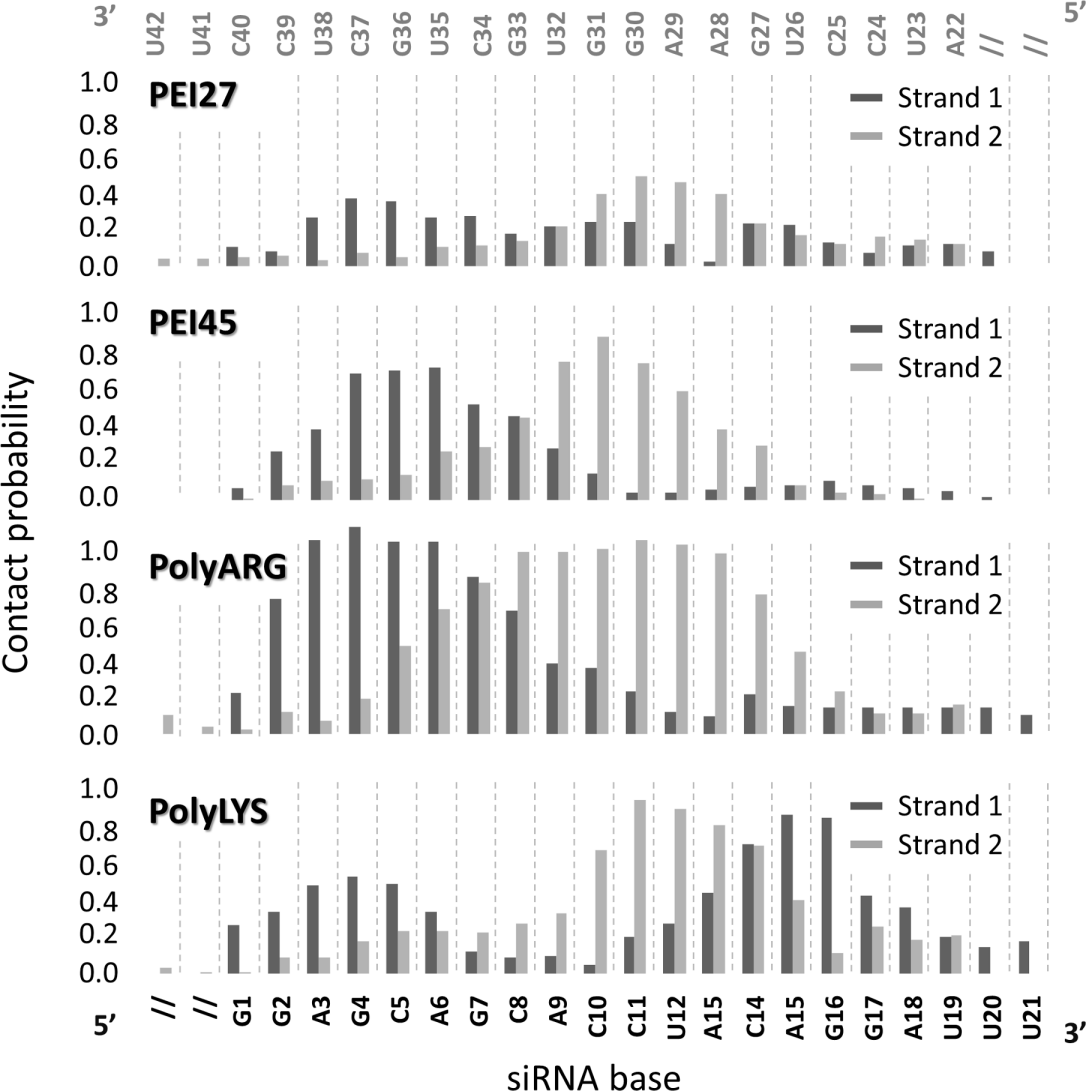
Nucleotides that are mainly responsible for siRNA-polycation interaction have been identified by contact probability plots [71] in case of PEI27, PEI45, polyARG and polyLYS systems.

**Fig 3:**
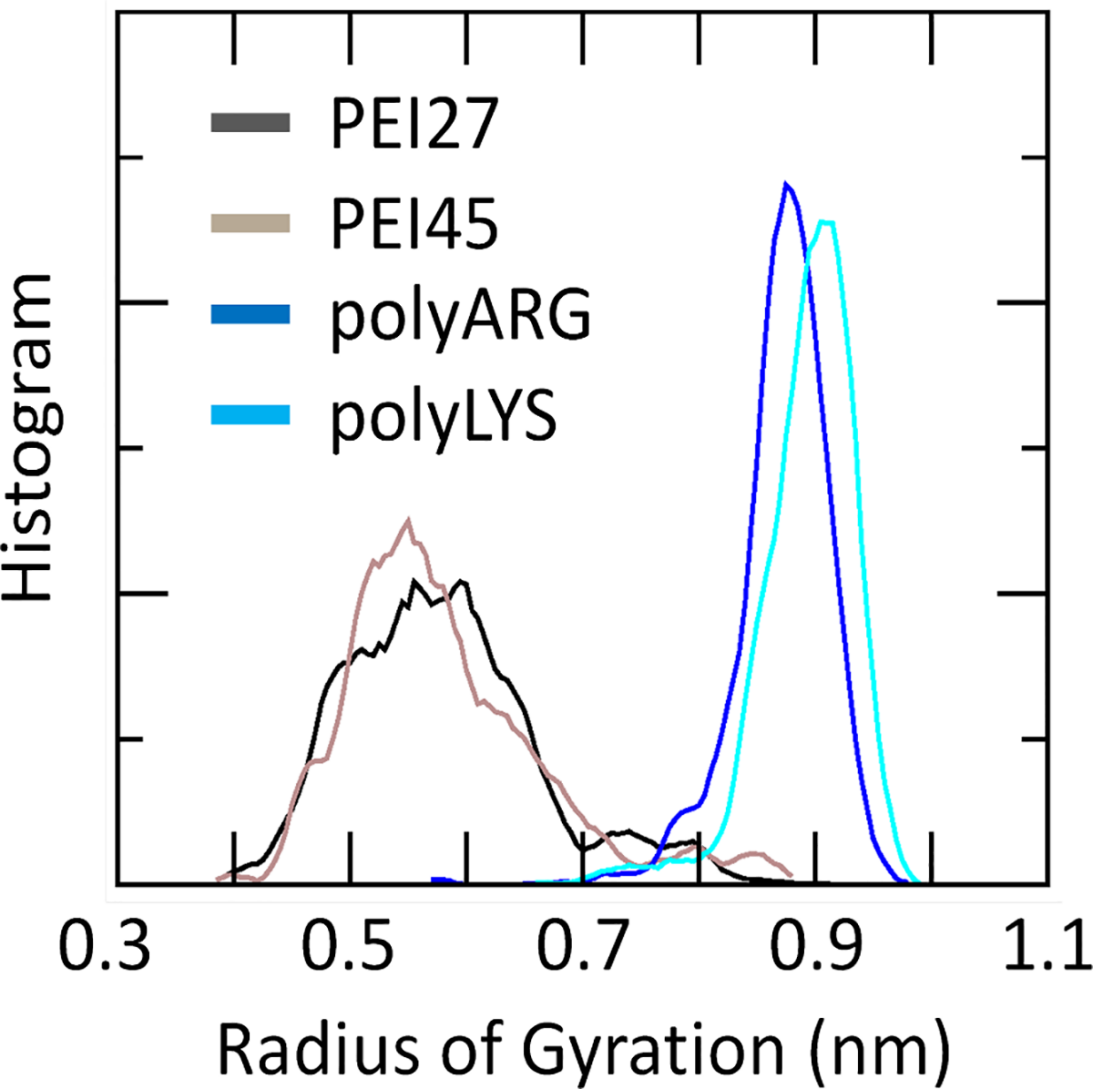
Distribution plot of the Radius of Gyration (RG) of polyARG, polyLYS, PEI27 and PEI45, respectively. The computational data were taken from the last 20 ns of the Replica Exchange Molecular Dynamics trajectory at 300K, for each molecular system.

REMD trajectory at 300K were analyzed in order to identify the nucleotides that are mainly responsible for siRNA-polycation interaction by contact probability plots. Nucleotides A28, A29, G30, G31 and U32 are strongly involved in the interaction with polyARG, polyLYS and PEI45 polymers, as demonstrated by the high contact probability values (Fig 2). Moreover, an additional common binding site involved nucleotides A3, G4, C5, A6, and G7. Lower contact probability values were detected in case of siRNA-PEI27, which is a sign of weaker electrostatic attraction caused by fewer positively charged amine groups available for the interaction. Polycation-siRNA interaction is strongly modulated by the chain flexibility, influencing the number of contacts during the binding phenomenon. REMD trajectory at 300K was analyzed in order to calculate the distribution plot of the Radius of Gyration (RG) of PEI27 and PEI45, polyARG, and polyLYS, respectively (Fig 3). PEI27 and PEI45 are the most compact structures, as demonstrated by the Radius of Gyration (RG) values of 5.9±0.8Å and 5.7±0.9Å, respectively, when compared to polyARG and polyLYS (RG_polyARG_ = 8.7±0.4Å, RG_polyLYS_ = 8.9±0.4Å). In addition, RG_polyARG_ and RG_polyLYS_ are characterized by smaller fluctuations, which implies that the polyARG and polyLYS undergo very little deformation during the simulations. This is a reasonable result considering both the high stiffness of the peptide bond and the steric hindrance of the amino acid side chain. On the contrary, PEI27 and PEI45 RG demonstrated a higher degree of flexibility. Moreover, the charge density loss from PEI45 to PEI27 has little influence on polymer flexibility.

**Fig 4:**
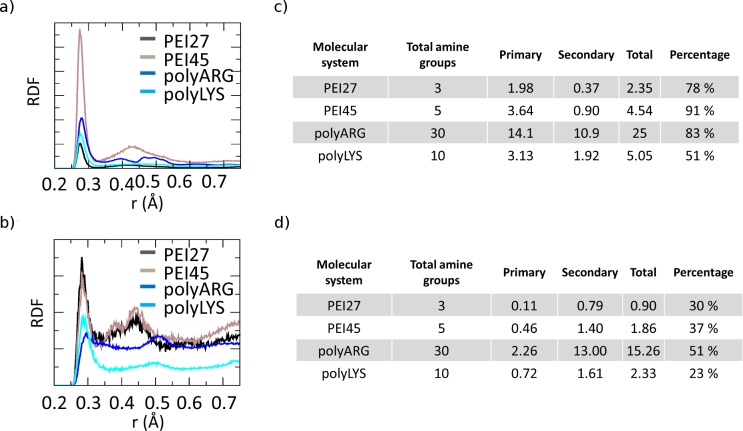
a) Radial distribution function (RDF) of polycation amine groups around O1P and O2P siRNA atoms for each molecular system reported in figure legend. Only charged amine groups are considered for the calculation. b) Average number of amine groups of each polycationic chain interacting with siRNA O1P and O2P atoms averaged over the last 20 ns of each Replica Exchange Molecular Dynamics trajectory at 300K. c) Radial distribution function (RDF) of polycation amine groups around electronegative atoms in siRNA grooves for each molecular system reported in figure legend. Only charged amine groups are considered for the calculation. The siRNA base edges that could participate in hydrogen bonds are N6 and N7 of adenine, O4 of uracil, O6 and N7 of guanine, and N4 of cytosine d) Average number of amine groups of each polycationic chain interacting with electronegative atoms in siRNA groves averaged over the last 20 ns of each Replica Exchange Molecular Dynamics trajectory at 300K.

REMD trajectory at 300K of each molecular system was analyzed to compute the RDF of the polycationic amine nitrogen atoms around O1P and O2P siRNA atoms (Fig 4A), in order to investigate the nature of the interactions between protonated amine groups and siRNA phosphate group oxygen atoms, as previously described [19,20,49]. The plots reported in Fig 4A are characterized by two different peaks at 2.7 Å and 4.3 Å, in agreement with previous computational observations [20]. The first peak is related to the primary interaction resulting from the hydrogen bond between the amine hydrogen atoms and the phosphate oxygen, while the second peak indicates the water-mediated hydrogen bonding. With the aim of quantifying the significant differences observed in Fig 4A in terms of peak heights, the contacts between the amine nitrogen atoms involved in primary or secondary interactions with the phosphate oxygens, were calculated over the last 20 ns of REMD trajectory at 300K, as shown in Fig 4A and 4B. Due to their highly flexible nature, PEI27 and PEI45 are able to interact with a high number of amine groups (77% and 91%) through their phosphate groups, demonstrating the alignment of the polycation backbone with the siRNA phosphate groups in the major grooves, in particular for PEI45. In contrast, polyLYS shows fewer positively charged groups in contact with the siRNA phosphate groups (51%). Despite its molecular stiffness, polyARG interacts with the electronegative atoms in the siRNA backbone (83%), due to its guanidinium group, which allows interactions in three possible directions through its three asymmetrical nitrogen atoms [81]. Aside from the specific interaction with the phosphate groups, PEI45 and polyARG also interact with electronegative atoms in siRNA major grooves with a high percentage of the available amine groups (37% and 51%, respectively), as shown in Fig 4C and 4D. In case of polyLYS the scenario is partially different. Despite the small number of positively charged groups interacting with the siRNA phosphate groups (51%), polyLYS also shows weak interaction with the electronegative atoms of major siRNA grooves (Fig 4D), suggesting that some amino acids are relatively far from the siRNA helix, in agreement with previous computational data [20].

### SiRNA-polycations free energy landscape

**Fig 5:**
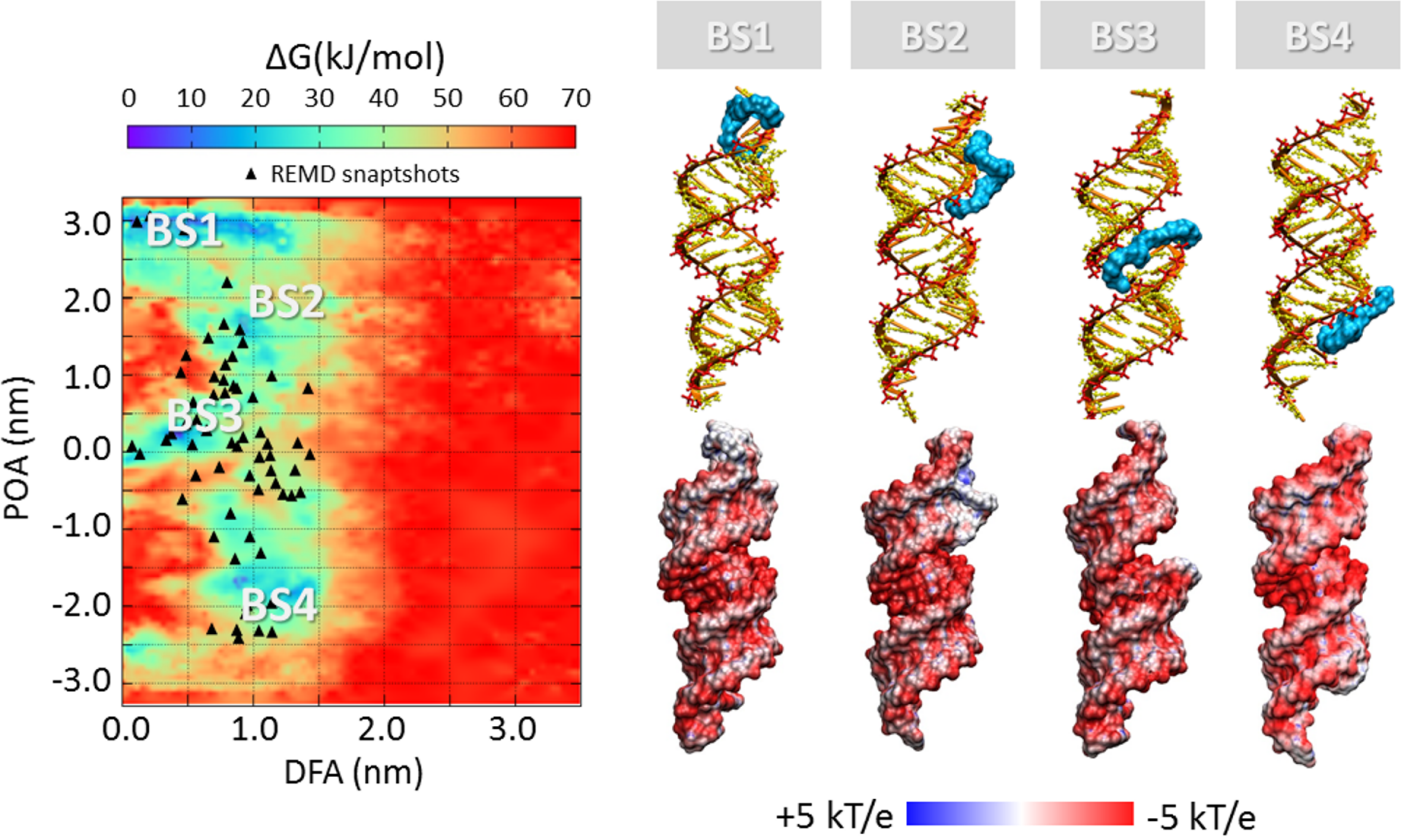
Free energy profile (kJ/mol) of siRNA-PEI27 interaction represented as function of siRNA-polycation distance from the major inertia axis of the target siRNA (distance from axis-DFA) and the projection of the siRNA-polymer distance on the major inertia axis of the target siRNA (projection on axis-POA). The free energy minima computed by metadynamics are compared with the system configurations sampled by REMD simulations (black triangle). The deepest binding site is highlighted with BS1. The visual inspection of each binding site is reported together with the corresponding electrostatic map (right). Potential isocontours are shown at +5kT/e (blue) and -5kT/e (red) and obtained by solution of the LPBE at 150 mM ionic strength with a solute dielectric of 4 and a solvent dielectric of 78.4.

**Fig 6:**
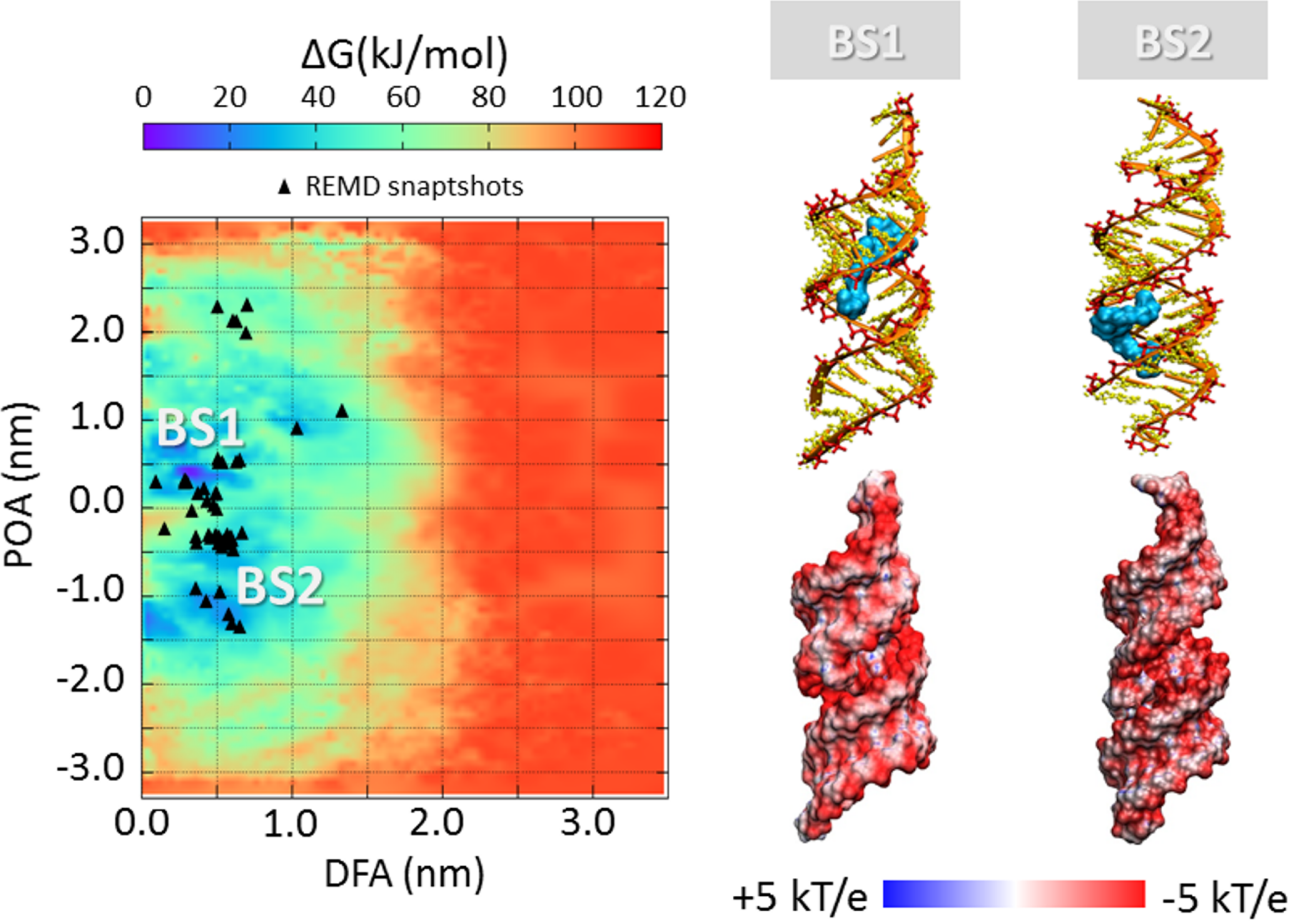
Free energy profile (kJ/mol) of siRNA-PEI45 interaction represented as function of siRNA-polycation distance from the major inertia axis of the target siRNA (distance from axis-DFA) and the projection of the siRNA-polymer distance on the major inertia axis of the target siRNA (projection on axis-POA). The free energy minima computed by metadynamics are compared with the system configurations sampled by REMD simulations (black trianle). The deepest binding site is highlighted with BS1. The visual inspection of each binding site is reported together with the corresponding electrostatic map (right). Potential isocontours are shown at +5kT/e (blue) and -5kT/e (red) and obtained by solution of the LPBE at 150 mM ionic strength with a solute dielectric of 4 and a solvent dielectric of 78.4.

**Fig 7:**
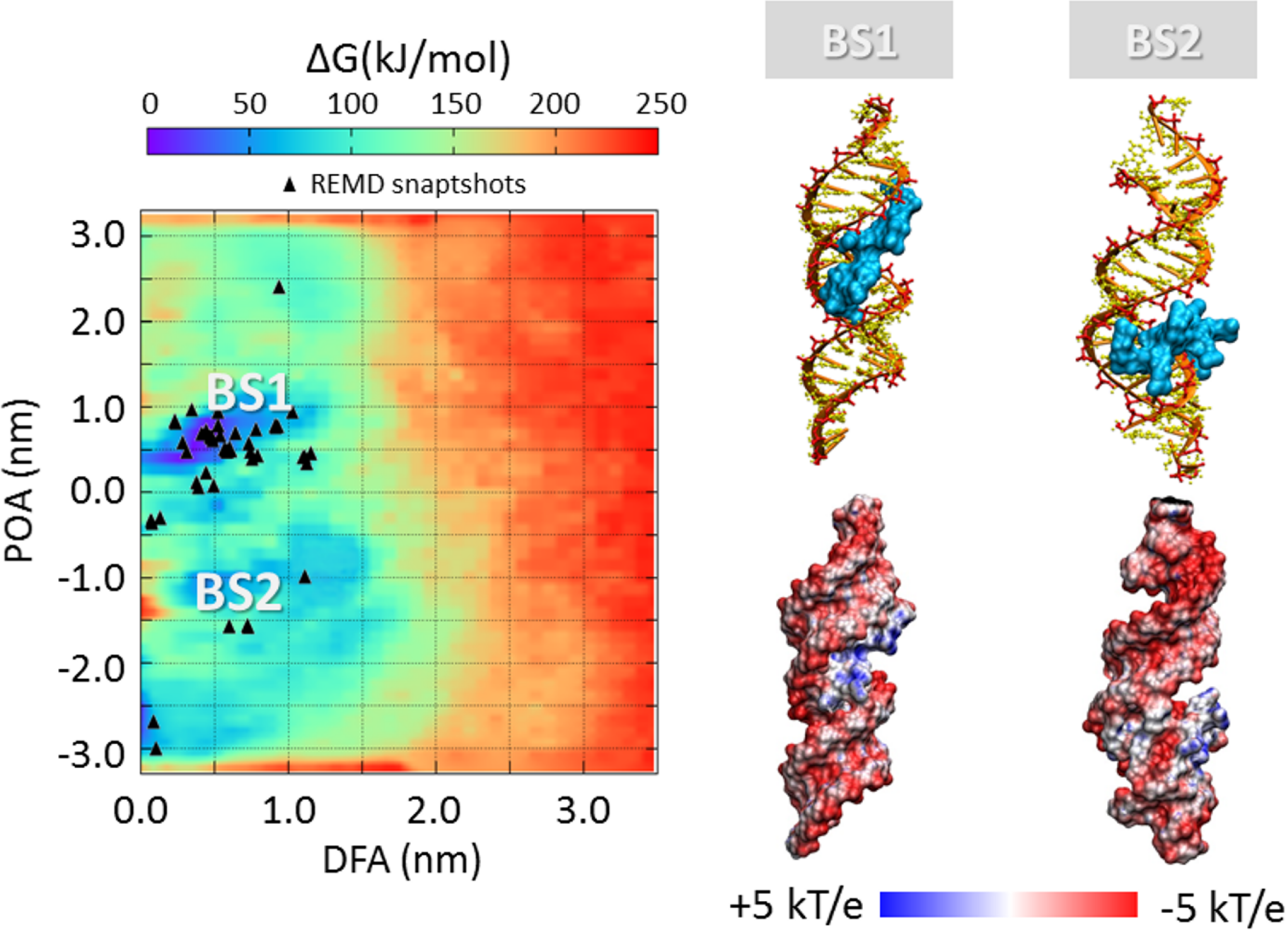
Free energy profile (kJ/mol) of siRNA-polyARG interaction represented as function of siRNA-polycation distance from the major inertia axis of the target siRNA (distance from axis-DFA) and the projection of the siRNA-polymer distance on the major inertia axis of the target siRNA (projection on axis-POA). The free energy minima computed by metadynamics are compared with the system configurations sampled by REMD simulations (black triangle). The deepest binding site is highlighted with BS1. The visual inspection of each binding site is reported together with the corresponding electrostatic map (right). Potential isocontours are shown at +5kT/e (blue) and -5kT/e (red) and obtained by solution of the LPBE at 150 mM ionic strength with a solute dielectric of 4 and a solvent dielectric of 78.4.

**Fig 8:**
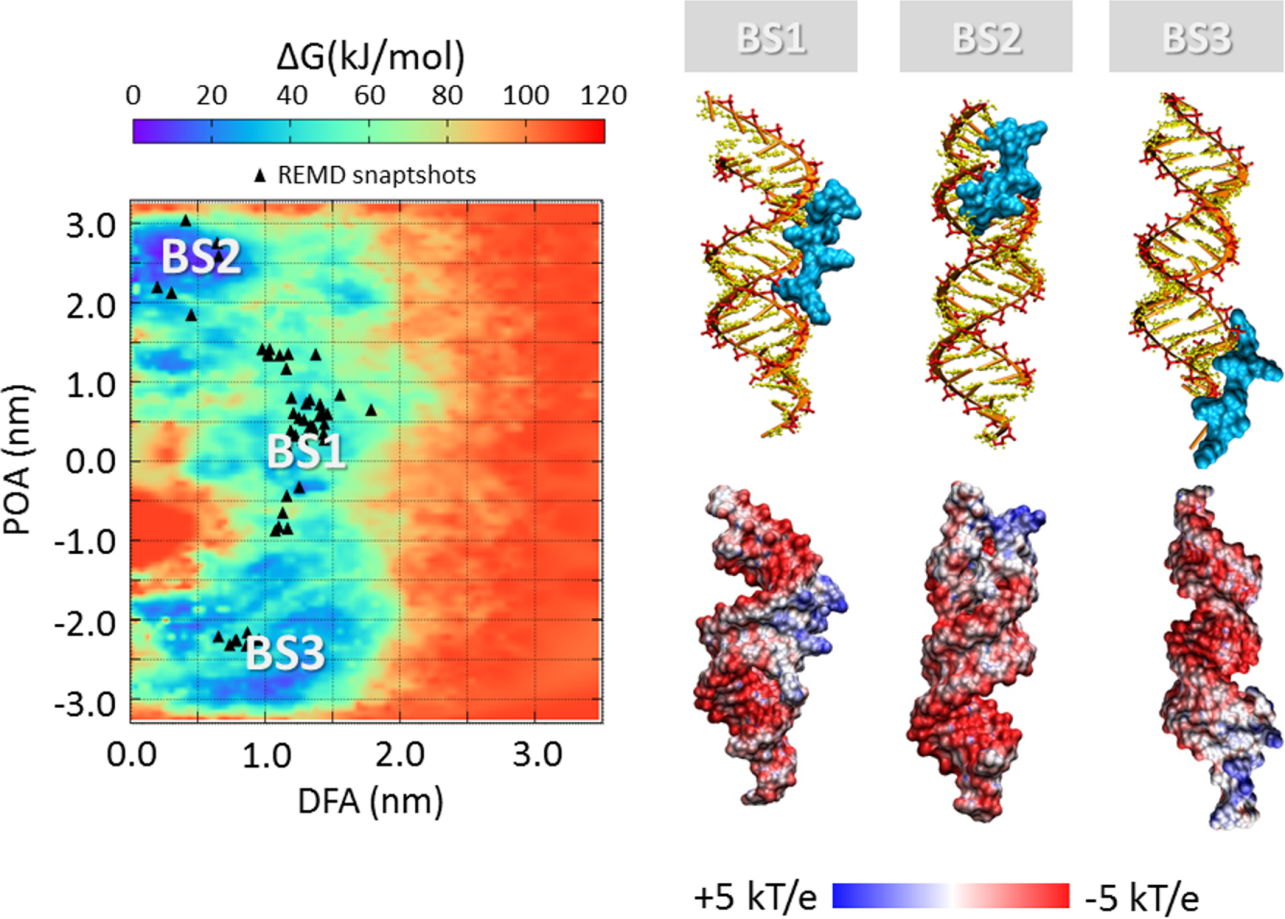
Free energy profile (kJ/mol) of siRNA-polyLYS interaction represented as function of siRNA-polycation distance from the major inertia axis of the target siRNA (distance from axis-DFA) and the projection of the siRNA-polymer distance on the major inertia axis of the target siRNA (projection on axis-POA). The free energy minima computed by metadynamics are compared with the system configurations sampled by REMD simulations (black triangle). The deepest binding site is highlighted with BS1. The visual inspection of each binding site is reported together with the corresponding electrostatic map (right). Potential isocontours are shown at +5kT/e (blue) and -5kT/e (red) and obtained by solution of the LPBE at 150 mM ionic strength with a solute dielectric of 4 and a solvent dielectric of 78.4.

The process of polycation unbinding from the siRNA displays interesting differences when investigated by a metadynamics approach. An overall picture of the siRNA-polycations free energy landscape is provided in Fig 5, Fig 6, Fig 7 and Fig 8, showing the 2D color maps of the interaction free energy in case of siRNA-PEI27, siRNA-PEI45, siRNA-polyARG, and siRNA-polyLYS, respectively. For each case, the free energy surface was represented as a function of two collective variables: a) the siRNA-polycation distance from the major inertia axis of the target siRNA (DFA), and b) the projection of the polymer-siRNA distance on the major inertia axis of the target siRNA (DOA). The first difference observed for the investigated systems concerns the polymer binding free energy. PolyARG shows the best binding affinity (ΔG_polyARG_ = 250 ± 5 kJ/mol) compared to polyLYS (ΔG_polyLYS_ = 120 ± 2 kJ/mol), PEI45 (ΔG_PEI45_ = 120 ± 2 kJ/mol), and PEI27 (ΔG_PEI27_ = 70 ± 1 kJ/mol), due to the high number of amine groups available for the interaction with siRNA. The net charge loss moving from PEI45 to PEI27 results in a decreased binding free energy, lowering the energetic cost for the escape of PEI27 from its binding sites. Moreover, few differences between the first binding pose of PEI45 (Fig 6) and PEI27 (Fig 5) can be noticed. In detail, the PEI45 binding sites are “sandwich-like” conformations inside the major grooves (BS1-Fig 5B). On the other side, PEI27 shows a hight degree of motility, allowing also the polycation interaction with the siRNA minor grooves. This evidence highlights the effect of the protonation ratio in modifying not only the binding affinity but also the binding conformation on the siRNA target. These outcomes are in line with the above mentioned ability of PEI45 to interact with siRNA phosphate groups via a high number of amine groups (Fig 4).

In case of the siRNA-polyARG system, both energy basins (Fig 7) correspond to major groove binding conformations, characterized by the polymer in close contact with siRNA phosphate groups. Despite the polyARG steric hindrance, the positively charged guanidinium moiety is able to interact with the siRNA phosphate groups in three possible directions through its three asymmetrical nitrogen atoms, allowing the alignment of polyARG with the siRNA backbone. By contrast, lysine moieties contain a positively charged amino group that forms only one hydrogen bond with anionic phosphate groups in a specific direction in the space, leading to the inability of polyLYS to maximize the electrostatic interactions with the siRNA backbone. As a consequence, the first polyLYS binding site (BS1-Fig 8) is characterized by increased DFA distance at the energy minimum (DFA_BS1-polyLYS_ = 1.3 nm) compared to polyARG (DFA_BS1-polyARG_ = 0.50 nm), given that some amino acids are located relatively far from the siRNA helix.

In all cases, the free energy minima are expected to match the energetically most favorable molecular system configurations. To further verify the stability of each siRNA-polycation binding conformation obtained by Metadynamics, it is interesting to compare the free energy map with the molecular configurations sampled by REMD (Fig 5, Fig 6, Fig 7 and Fig 8). As expected, free energy minima computed by metadynamics lie in regions regularly sampled by REMD, confirming the ability of the metadynamics simulations in correctly identifying the free energy well.

### Charge neutralization and electrostatic potential

The most representative binding conformations, found at the free energy minima, were further investigated by calculating the electrostatic potential of the resulting complex. The most obvious feature is the overall negative electrostatic potential of the siRNA, which plays a significant role in the interaction with siRNA-associated macromolecules (Fig 5, Fig 6, Fig 7 and Fig 8). Quantitative examination of the electrostatic data shows interesting differences in the polymer binding regions. While PEI27 and PEI45 (Fig 5 and Fig 6) were able to neutralize the siRNA electrostatic potential in the binding region, polyARG (Fig 7) and in particular polyLYS (Fig 8) were surrounded by a positive potential, which may allow the formation of multivalent interactions with other siRNA molecules.

## Discussion

The purpose of the present study was to elucidate dominant pathways of the siRNA-polycations self-assembly process by enhanced sampling techniques. With the aim of investigating the effect of molecular geometry, polymer flexibility, and charge density on the siRNA-polycation free energy landscape, different polymers were selected for the calculation: polyLYS, polyARG, and PEI with the same number of monomer repeating units.

### Polycation flexibility, molecular geometry and siRNA charge neutralization

Polycation chain flexibility is known to play a pivotal role in siRNA-polycation interaction [19,50,82]. For this reason, the flexibility/rigidity ratio was considered as one of the five critical parameters (size, shape, surface chemistry, flexibility/rigidity, and molecular geometry) that must be taken into account at the nanoscale level for modelling a polymer carrier [83]. Recently, different PEI/DNA binding configurations for linear and branched PEI were observed, resulting from the difference in polymer flexibility [19]. Pavan et al. have demonstrated that rigid polycationic dendrimers are able to reorganize their peripheral groups to generate a large number of contacts with the nucleic acid [50]. They found that flexible dendrimers, originally conceived to create multivalent interactions with nucleic acids, generate only few contacts, revealing the role of molecular flexibility in the binding phenomenon. Here, we provided evidences that PEI polymers, regardless of their protonation ratio, are characterized by a high degree of flexibility at atomic scale compared to polyARG and polyLYS. This can be explained by both the high stiffness of the peptide bond and the steric hindrance of the amino-acid side chain. Thus, in case of polyARG and polyLYS chains, any large conformational change from the equilibrated arrangement will introduce a significant energy penalty. The flexibility loss moving from PEI to polyLYS results in a decreased ability of the polycation to align its backbone with the phosphate groups of the siRNA. As a result, polyLYS interacts with phosphate groups and some amino acids are relatively far from the siRNA helix (Fig 8), making themselves available for the interaction with other siRNA molecules. The multivalent character of polyLYS is also demonstrated by the quantitative examination of the electrostatic data presented in Fig 8, showing that polyLYS is able to over-compensate the siRNA electrostatic potential in the binding region. In case of the siRNA-polyARG interaction, the scenario is different. Despite its rigidity, polyARG is able to align its backbone with the siRNA major groove thanks to its molecular geometry. In detail, the positively charged guanidinium group allows for interactions in three possible directions through its three asymmetrical nitrogen atoms [81].

### Free energy landscape and impact for tailoring polymer design

The free energy estimation provided in the present work may be used within the design process of potent nucleic acids binders able to finally reach the best compromise between complexation stability and release ability. For example, tailoring for a strong siRNA-polycation complexation is important for the use of such complexes *in vivo*, where highly bound complexes have been shown to be more resilient against degradation [84]. On the one hand, a functional consequence of the siRNA-polycation enhanced binding affinity could be an improved gene expression due to increased cellular uptake and/or better protection against degradation [85]. On the other hand, a less stable siRNA-polymer interaction may lead to an increased release ability, making the nucleic acids more easily available inside the cells. In this view, molecular modeling represents a powerful tool to optimize the siRNA-polycationic chain interaction in order to optimize the complexation stability and release ability [15–18]. Here we demonstrated that polyARG has the best binding affinity compared to polyLYS, PEI45, and PEI27, due to the high number of amine groups available for the interaction with siRNA (Fig 5, Fig 6, Fig 7 and Fig 8). Moreover, the net charge loss moving from PEI45 to PEI27 results in a decreased binding free energy, lowering the energetic cost for the escape of PEI27 from its binding sites. Although the predominant PEI-siRNA interaction is expected involve the electronegative oxygen atoms on the siRNA backbone and protonated PEI nitrogens, our simulations also predict interactions with the siRNA base oxygens and nitrogens, implying siRNA groove binding of the PEI and polyARG, in agreement with other experimental and computational studies [19,40,86].

These results have several implications in the field of nucleic acid condensation [87], in particular for the difference in the ability of arginine and lysine peptides to compact DNA, which is a quite substantial issue. It was reported that the equilibrium spacing between DNA helices condensed by lysine are significantly larger than the inter-helical distances condensed by arginine peptides [88]. In detail, the equilibrium surface-surface distance between DNA helices increases by almost 50% with a change from polyarginine to polylysine [88]. Here, we showed that the first polyLYS binding site is characterized by an increased distance from the siRNA major axis of inertia when compared to polyARG, given that some amino acids are located relatively far from the siRNA helix. Moreover, we have identified significant differences between the polyLYS and polyARG binding mode, in contrast to the previous assumption of them being similar [88]. Our results are in line with a previously proposed model for polyARG interaction with nucleic acids [89], and show that the polyARG backbone is able to interact with the siRNA major groove, allowing the side chain guanidinium groups to bind to the neighboring phosphates along the strands by hydrogen bonding. By contrast, lysine moieties contain a positively charged amino group that forms only one hydrogen bond with anionic phosphate groups in a specific direction in the space, leading to the inability of polyLYS to maximize the electrostatic interactions with the siRNA backbone. As a consequence, the first polyLYS binding site is characterized by an increased distance from the siRNA major axis of inertia when compared to polyARG.

It is worth mentioning that the polycations simulated in this work are characterized by a low molecular weight (LMW). Although experiments have shown that polymers with higher molecular weights are more effective for gene delivery, the high toxicity of hight MW polymers, such as high MW PEI, limits their use in medical applications. Therefore more recent studies investigated the delivery of nucleic acids with modified LMW polymers [90,91], which encouraged the present computational study of siRNA interactions with the presented LMW polycations. Moreover, it is not practical to simulate high molecular weight polymers by MD, even with state-of-the-art enhanced sampling techniques [19–23,50]. Nevertheless, the obtained results with LMW polymers are expected to shed light on siRNA binding mechanism, considering the binding mechanism being similar for a polymer of higher molecular weight.

In conclusion, our work provides novel insight into the siRNA-polycation complexation mechanism, elucidating how polycation-siRNA binding modes and free energy landscapes are influenced by the physico-chemical properties of the interacting polymers. An exhaustive exploration of all the possible binding sites was provided by REMD and metadynamics, taking fully into account the siRNA flexibility together with the presence of explicit solvent and ions. These findings render our simulation protocol suitable for further investigations on polymers/nucleic-acids interaction to assist the design/development of potent and selective DNA/RNA drug carrier systems.

## Supporting information

S1 File The supporting information text contains details concerning applied procedures and validation checks. The file is organized in three main sections: S1.1 Replica Exchang Molecular Dynamics, S1.2. Metadynamics: Definition of the Collective Variables, S1.3. Reweighting algorithm procedure, and S1.4 Convergence of the Metadynamics free energy estimation.
(PDF)
