# Supplementary material for: Free energy landscape of siRNA-polycation complexation: Elucidating the effect of molecular geometry, polymer flexibility, and charge neutralization
Source: PLoS One. 2017 Oct 31;12(10):e0186816. doi: 10.1371/journal.pone.0186816 (PMC5663398; doi:10.1371/journal.pone.0186816)
Supplement: S1 File — The file is organized in three main sections: S1.1 Replica Exchang Molecular Dynamics, S1.2. Metadynamics: Definition of the Collective Variables, S1.3. Reweighting algorithm procedure, and S1.4 Convergence of the Metadynamics free energy estimation. (PDF) [file pone.0186816.s001.pdf]

SUPPORTING INFORMATION

S1.1 Replica Exchange Molecular Dynamics

Replica Exchange Molecular Dynamics (REMD) [1] was carried out to efficiently sample the polymer-RNA complex formation, following a computational procedure successfully applied in literature [2–6]. In detail, 128 replicas were simulated for temperatures ranging from 300 K to 530 K in NVT ensemble, obtaining a cumulative simulation time of 6.5 μs for each system. Simulated temperatures were distributed following the exponential spacing law as suggested in literature [7,8], in order to obtain a constant overlap of the potential energy distributions among temperatures (Figure S1). The exchange attempt time interval was set to 2 ps. At the end of the simulation, the resulting average exchange probability was 0.4.

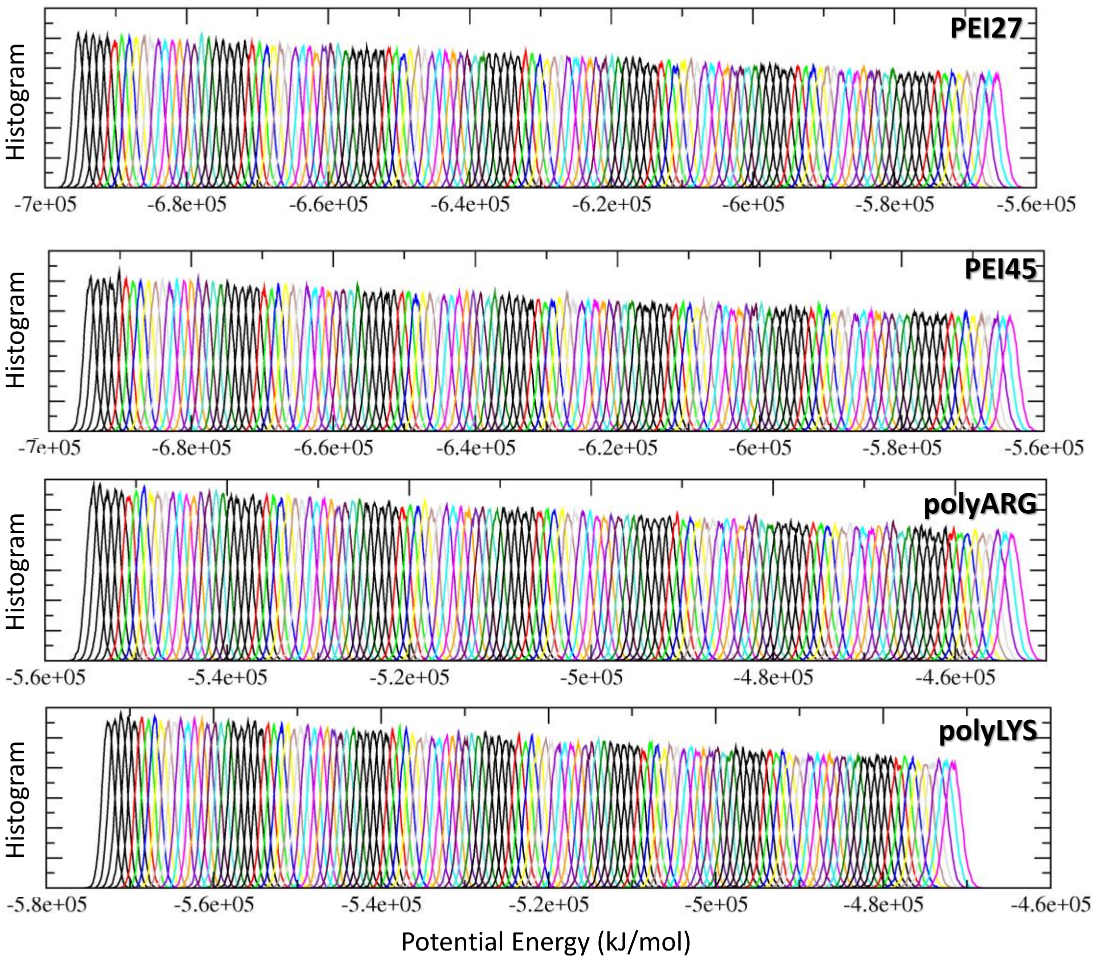

Figure S1. Potential energy distributions calculated for each replica of the REMD simulation in case of siRNA-PEI27, siRNA-PEI45, siRNA-polyARG and siRNA-polyLYS systems.

We have verified the convergence of the REMD simulation analyzing the distribution of the polymer distance from the siRNA major axis of inertia, calculated over different time windows: 15ns-20ns, 15ns-25ns, 15ns-30ns, 15ns-35ns, 15ns-40ns, 15ns-45ns, 15ns-50ns. It is interesting to notice that the differences between the distributions are negligible comparing the time windows 15-45ns and 15-50ns, demonstrating the convergence of the simulation. This let us think that incrementing the simulation time will not bring to any considerable alteration of the free energy profile.

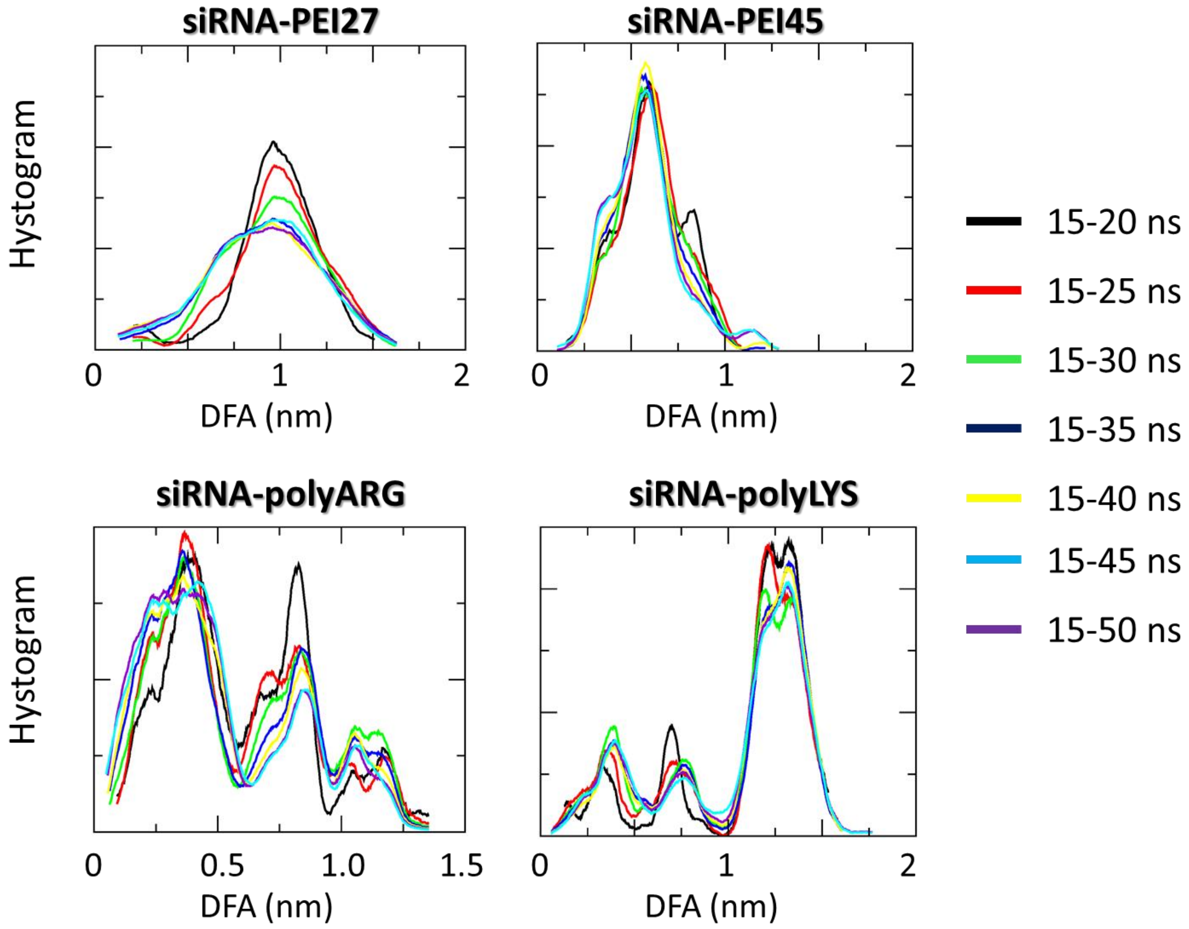

Figure 2. Distribution of the polymer distance from the siRNA major axis of inertia (DFA), calculated over different time windows: 15ns-20ns, 15ns-25ns, 15ns-30ns, 15ns-35ns, 15ns-40ns, 15ns-45ns, 15ns-50ns. It is interesting to notice that the differences between the distributions are negligible comparing the time windows 15-45ns and 15-50ns, demonstrating the convergence of the REMD simulation.

S1.2 Metadynamics: Definition of the Collective Variable

The free energy landscape representing the siRNA-polycationic chain interaction was investigated by means of Metadynamics (metaD) [9,10]. In metaD, an external history-dependent bias potential which is a function of a few selected collective variables (CVs) is added to the Hamiltonian of the system. This potential is conceived as a sum of Gaussians deposited along the system trajectory in

the CV space to discourage the system from revisiting configurations that have already been sampled. In the widely used well-tempered procedure [11] the bias deposition rate decreases over simulation time as follows:

$$F(s,t) = -\frac{T+\Delta T}{T} V(s,t), \quad \text{where } \gamma = -\frac{T+\Delta T}{T} \text{ is the bias factor}$$

A bias factor of 30, 50, 100, and 45 was used in case of siRNA-PEI27, siRNA-PEI45, siRNA-polyARG, and siRNA-polyLYS simulations. Gaussian hills are added at a constant time interval in the position explored by the system in the space of selected Collective Variables (CVs). Two CVs were used for each simulation: a) the distance between the COM of the polymer from the major inertia axis of the target siRNA (distance from axis-DFA); b) the projection of the COM of the polymer on the major inertia axis of the target siRNA (projection on axis-POA). Gaussian widths of 0.05 nm and 0.1 nm were used, for DFA and POA reaction coordinates, respectively. The gaussian width value was of the same order of magnitude as the standard deviation of the collective variable, calculated during unbiased simulations (Table S1).

Table S1. Standard deviation of the DFA and POA during the unbiased MD simulations.

UNBIASED MD SIMULATION	STANDARD DEVIATION OF DFA (nm)	STANDARD DEVIATION OF POA (nm)
**siRNA-pei27**	0.09	0.24
**siRNA-pei45**	0.08	0.15
**siRNA-polyLYS**	0.05	0.12
**siRNA-polyARG**	0.06	0.10

S1.3 Reweighting algorithm procedure

The reconstruction of the free-energy surface was performed by the reweighting algorithm procedure [12], allowing the identification of the main polymer binding sites together with the lowest energy conformations as function of four CVs:

i. The distance between the COM of the polymer from the major inertia axis of the target siRNA (distance from axis-DFA)
ii. The projection of the COM of the polymer on the major inertia axis of the target siRNA (projection on axis-POA)

Each CV has demonstrated to be helpful in describing the binding events in the context of protein-nucleic acids complexes by metadynamics [13–16].

S1.4 Convergence of the metadynamics free energy estimation

The convergence of the Metadynamics simulation was demonstrated by following a well-established computational procedure performed in a recent work [17], which require to check i) recrossing events between low energy states and ii) fluctuations of the free energy difference between the low-energy states around a specific value.

i) Several recrossing events between low energy states can be identified analyzing the time evolution of the CVs (Figure S3, Figure S4, Figure S5, Figure S6). These events lead to the convergence in the estimation of the protein free energy state

ii) The free energy difference between low energy states at different times along the simulation (Figure S3f, Figure S4f, Figure S5f, Figure S6f) was calculated to assess the convergence. In all cases, the estimated free energy profile is reasonably stable in the last 100 ns. It is worth mentioning that the uncertainty, calculated as the SD from the asymptotic value of the free energy obtained from the last part of the simulation, does not take into account the force-field inaccuracy.

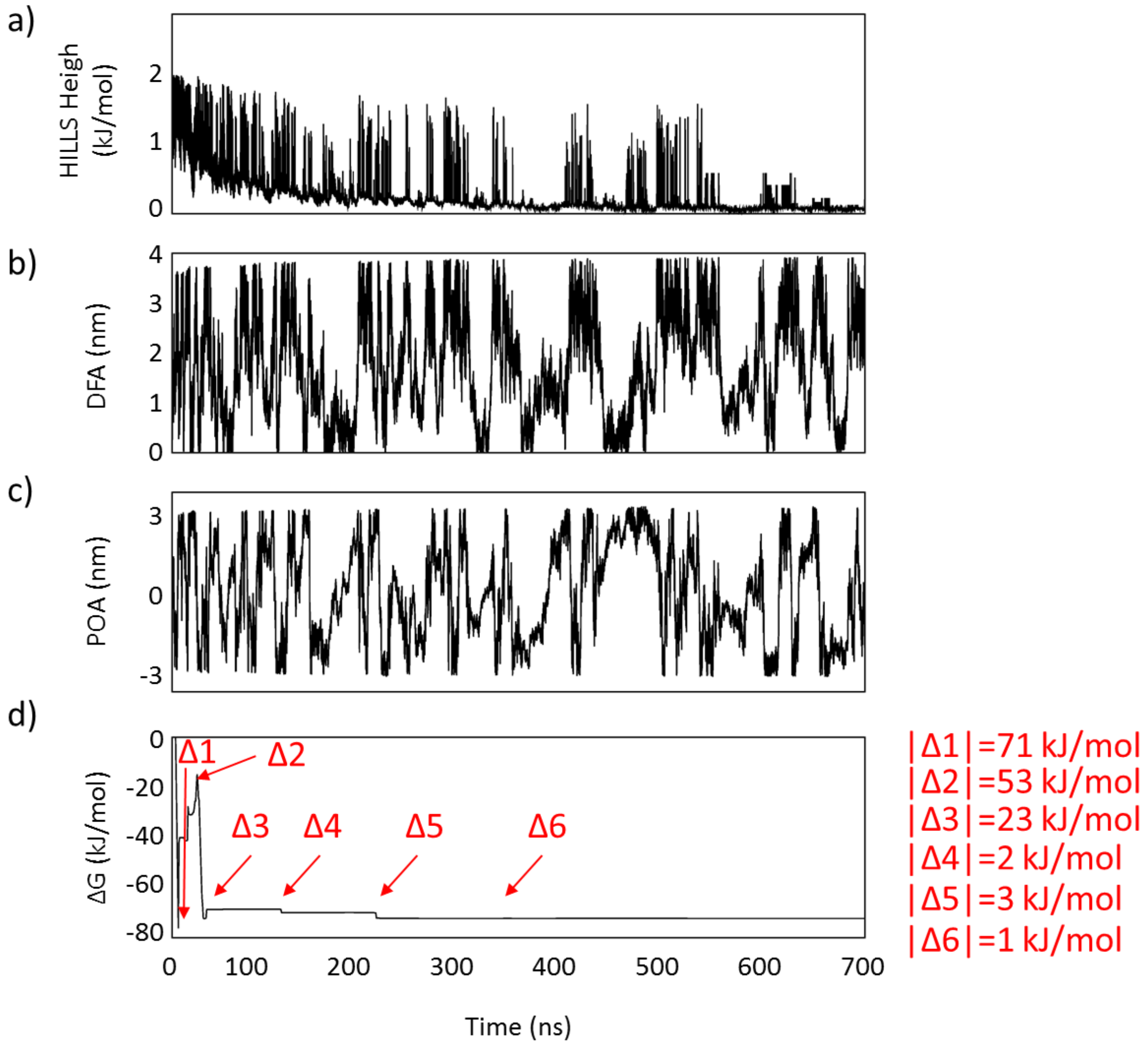

Figure S3. Convergence of the siRNA-PEI27 metadynamics simulation. a) Plot of the Gaussian Height added to the system along the Metadynamics simulation. b) Plot of the DFA collective variable along the metadynamics simulation. c) Plot of the POA collective variable along the metadynamics simulation. Several recrossing events between the low energy states can be identified. d) ΔG is calculated between the deepest bound state (BS1) and the siRNA-polymer unbound state to demonstrate the convergence of the free energy estimation. The uncertainty, calculated as the SD from the asymptotic value of the free energy obtained from the last part of the simulation is 1.0 kJ/mol.

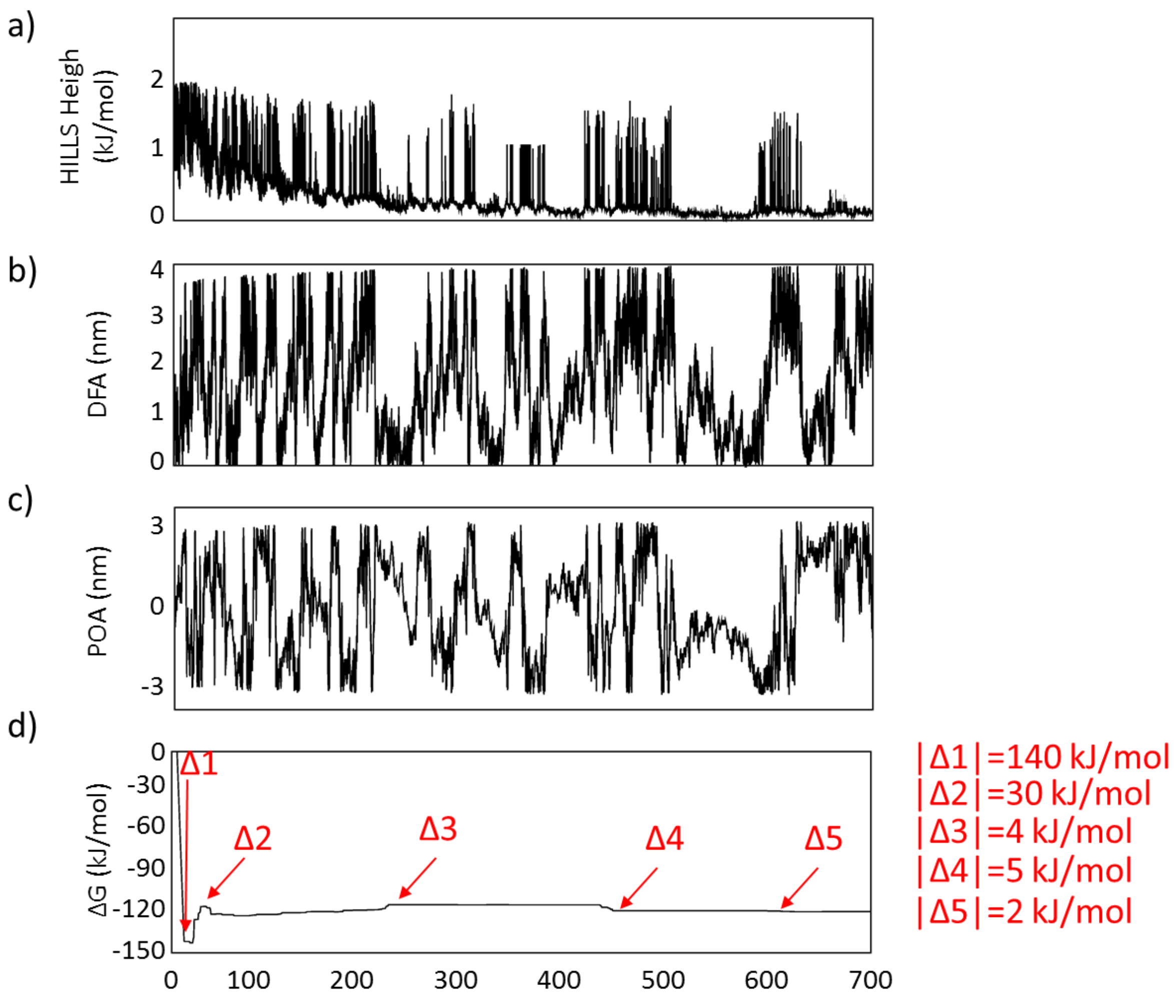

Figure S4. Convergence of the siRNA-PEI45 metadynamics simulation. a) Plot of the Gaussian Height added to the system along the Metadynamics simulation. b) Plot of the DFA collective variable along the metadynamics simulation. c) Plot of the POA collective variable along the metadynamics simulation. Several recrossing events between the low energy states can be identified. d) ΔG is calculated between the deepest bound state (BS1) and the siRNA-polymer unbound state to demonstrate the convergence of the free energy estimation. The uncertainty, calculated as the SD from the asymptotic value of the free energy obtained from the last part of the simulation is 2.0 kJ/mol.

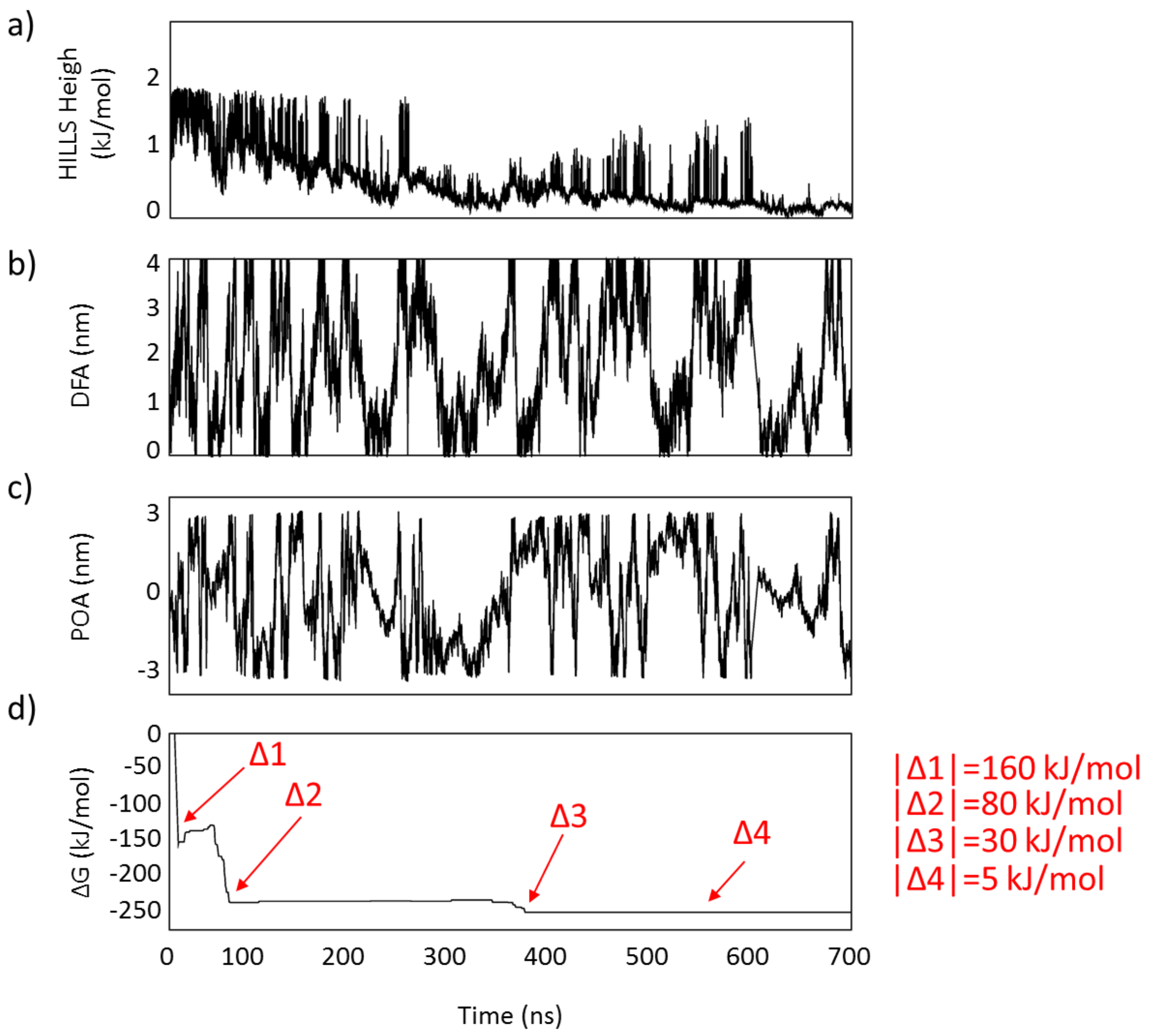

Figure S5. Convergence of the siRNA-polyARG metadynamics simulation. a) Plot of the Gaussian Height added to the system along the Metadynamics simulation. b) Plot of the DFA collective variable along the metadynamics simulation. c) Plot of the POA collective variable along the metadynamics simulation. Several recrossing events between the low energy states can be identified. d) ΔG is calculated between the deepest bound state (BS1) and the siRNA-polymer unbound state to demonstrate the convergence of the free energy estimation. The uncertainty, calculated as the SD from the asymptotic value of the free energy obtained from the last part of the simulation is 5.0 kJ/mol.

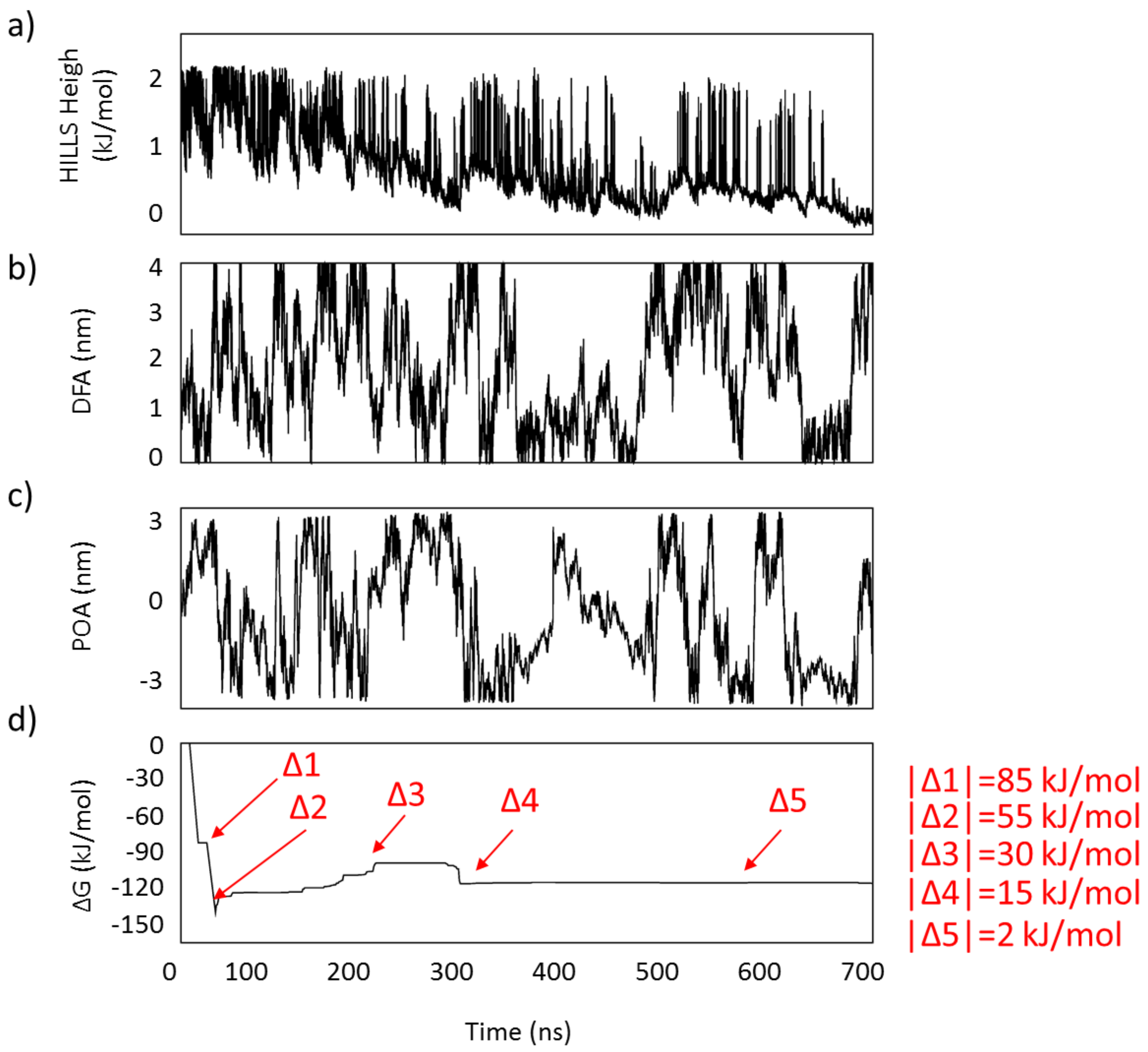

Figure S6. Convergence of the siRNA-polyLYS metadynamics simulation. a) Plot of the Gaussian Height added to the system along the Metadynamics simulation. b) Plot of the DFA collective variable along the metadynamics simulation. c) Plot of the POA collective variable along the metadynamics simulation. Several recrossing events between the low energy states can be identified. d) ΔG is calculated between the deepest bound state (BS1) and the siRNA-polymer unbound state to demonstrate the convergence of the free energy estimation. The uncertainty, calculated as the SD from the asymptotic value of the free energy obtained from the last part of the simulation is 2.0 kJ/mol.

References

S1. Sugita Y, Okamoto Y. Replica-exchange molecular dynamics method for protein folding. Chem Phys Lett. 1999;314: 141–151. doi:10.1016/S0009-2614(99)01123-9

2. Zerze GH, Miller CM, Granata D, Mittal J. Free Energy Surface of an Intrinsically Disordered Protein: Comparison between Temperature Replica Exchange Molecular Dynamics and Bias-Exchange Metadynamics. J Chem Theory Comput. 2015;11: 2776–2782. doi:10.1021/acs.jctc.5b00047

3. Nguyen PH, Stock G, Mittag E, Hu CK, Li MS. Free energy landscape and folding mechanism of a β-hairpin in explicit water: A replica exchange molecular dynamics study. Proteins Struct Funct Genet. 2005;61: 795–808. doi:10.1002/prot.20696

4. Bergonzo C, Henriksen NM, Roe DR, Swails JM, Roitberg AE, Cheatham TE.

Multidimensional Replica Exchange Molecular Dynamics Yields a Converged Ensemble of an RNA Tetranucleotide. J Chem Theory Comput. 2014;10: 492–499. doi:10.1021/ct400862k
5. Deriu MA, Grasso G, Tuszynski JA, Massai D, Gallo D, Morbiducci U, et al. Characterization of the AXH domain of Ataxin-1 using enhanced sampling and functional mode analysis. Proteins Struct Funct Bioinforma. 2016;84: 666–673. doi:10.1002/prot.25017
6. Grasso G, Deriu MA, Tuszynski JA, Gallo D, Morbiducci U, Danani A. Conformational fluctuations of the AXH monomer of Ataxin-1. Proteins Struct Funct Bioinforma. 2016;84: 52–59. doi:10.1002/prot.24954
7. Denschlag R, Lingenheil M, Tavan P. Optimal temperature ladders in replica exchange simulations. Chem Phys Lett. 2009;473: 193–195. doi:10.1016/j.cplett.2009.03.053
8. Lingenheil M, Denschlag R, Mathias G, Tavan P. Efficiency of exchange schemes in replica exchange. Chem Phys Lett. 2009;478: 80–84. doi:10.1016/j.cplett.2009.07.039
9. Barducci A, Bonomi M, Parrinello M. Metadynamics. Wiley Interdisciplinary Reviews: Computational Molecular Science. 2011. pp. 826–843. doi:10.1002/wcms.31
10. Laio A, Parrinello M. Escaping free-energy minima. Proc Natl Acad Sci U S A. 2002;99: 12562–12566. doi:10.1073/pnas.202427399
11. Barducci A, Bussi G, Parrinello M. Well-Tempered Metadynamics: A Smoothly Converging and Tunable Free-Energy Method. Phys Rev Lett. 2008;100: 20603. doi:10.1103/PhysRevLett.100.020603
12. Bonomi M, Barducci A, Parrinello M. Reconstructing the equilibrium boltzmann distribution from well-tempered metadynamics. J Comput Chem. 2009;30: 1615–1621. doi:10.1002/jcc.21305
13. Furini S, Domene C. DNA Recognition Process of the Lactose Repressor Protein Studied via Metadynamics and Umbrella Sampling Simulations. J Phys Chem B. 2014;118: 13059–13065. doi:10.1021/jp505885j
14. Coletta A, Desideri A. Role of the protein in the DNA sequence specificity of the cleavage site stabilized by the camptothecin topoisomerase IB inhibitor: a metadynamics study. Nucleic Acids Res. 2013;41: 9977–9986. doi:10.1093/nar/gkt790
15. Vargiu A V., Ruggerone P, Magistrato A, Carloni P. Dissociation of minor groove binders from DNA: insights from metadynamics simulations. Nucleic Acids Res. 2008;36: 5910–5921. doi:10.1093/nar/gkn561
16. Pérez-Villa A, Darvas M, Bussi G. ATP dependent NS3 helicase interaction with RNA: insights from molecular simulations: Table 2. Nucleic Acids Res. 2016;44: 2975–2975. doi:10.1093/nar/gkv1378
17. Limongelli V, Bonomi M, Parrinello M. Funnel metadynamics as accurate binding free-energy method. Proc Natl Acad Sci U S A. 2013;110: 6358–6363. doi:10.1073/pnas.1303186110
